# Evaluation of the Effect of Topical Prostaglandin Analog Treatment on Orbital Structures in Open-Angle Glaucoma with Computed Tomography

**DOI:** 10.3390/jcm13195808

**Published:** 2024-09-28

**Authors:** Berire Şeyma Durmuş Ece, Zübeyir Yozgat, Hüseyin Bayramlı, Bunyamin Ece, Sonay Aydin

**Affiliations:** 1 Department of Ophthalmology, Kastamonu University, Kastamonu 37150, Turkey; zyozgat@kastamonu.edu.tr (Z.Y.); hbayramli@kastamonu.edu.tr (H.B.); 2 Department of Radiology, Kastamonu University, Kastamonu 37150, Turkey; bunyaminece@kastamonu.edu.tr; 3 Department of Radiology, Erzincan Binali Yıldırım University, Erzincan 24100, Turkey; sonay.aydin@erzincan.edu.tr

**Keywords:** glaucoma, prostaglandin analogs, prostaglandin-associated periorbitopathy, computed tomography, enophthalmos

## Abstract

**Background/Objectives**: This study aims to evaluate the computed tomography (CT) scans of glaucoma patients using prostaglandin analogs (PGA) in one eye, investigate findings associated with prostaglandin-associated periorbitopathy (PAP), and compare these findings with those of the contralateral eyes. **Methods**: Patients with open-angle glaucoma who had CT images of the orbital region taken for another reason at least one month after starting PGA treatment in one eye were included in the study. Enophthalmos measurements from thin-slice CT images, along with 3D volume measurements of orbital fat tissue, periorbital muscles, and the optic nerve, were performed. Ophthalmological examination findings and treatment information were collected. The values were compared with those of the contralateral eyes of the same patients not using PGA. Intraclass correlation coefficients (ICCs) were computed to evaluate measurement repeatability. **Results**: Forty patients were included in the study. Among them, 29 (72.5%) used latanoprost, 9 (22.5%) used bimatoprost, and 2 (5%) used travoprost. The mean enophthalmos values on the treated side (15.5 ± 2.0 mm) were lower than on the untreated side (16.1 ± 1.4 mm), but this difference was not statistically significant (*p* = 0.07). In 29 patients (72.5%), enophthalmos measurements were smaller on the treated side, with 7 patients (17.5%) showing a difference of 2 mm or more. No significant correlation was found between the duration of PGA use and enophthalmos measurements (*p* = 0.768 r = −0.048). Additionally, no significant differences were found in orbital fat volume, total extraocular muscle volume, and optic nerve volume (*p* > 0.05). ICC values demonstrated excellent reliability (ICC > 0.75) for all measurements. **Conclusions**: We did not find significant differences in enophthalmos measurements, orbital fat volume, total muscle volume, and optic nerve volume between the PGA-treated and untreated eyes.

## 1. Introduction

Glaucoma is an optic neuropathy characterized by the progressive degeneration of retinal ganglion cells. It affects over 70 million people worldwide and is the leading cause of irreversible blindness. The most significant known risk factor in the development of glaucoma is elevated intraocular pressure (IOP). Reducing IOP is the only proven method for effectively treating glaucoma. The primary goal of treatment is to reach the target IOP with the fewest medications and minimal side effects [1]. Prostaglandin analog (PGA) lower IOP by approximately 20–35% via increasing aqueous outflow through the uveoscleral pathway. Due to their effective IOP reduction, minimal systemic side effects, and once-daily application, PGAs are the first line of medical treatment [2]. Despite their high systemic safety profile, chronic use of PGA can cause side effects affecting the ocular surface and periocular region. These local side effects include conjunctival hyperemia, hypertrichosis, iris pigmentation, periocular pigmentation, orbital fat atrophy, and changes in the adnexal tissue. The general term used to describe the eyelid and orbital changes associated with topical PGA use is prostaglandin-associated periorbitopathy (PAP) [3].

The first report related to PAP was described in 2004 as deepening of the upper eyelid sulcus (DUES) in patients using bimatoprost. The authors associated this condition with the potential effects of bimatoprost on Müller’s muscle [4]. The prevalence of DUES after at least three months of treatment is 44%, and its incidence ranges from 25 to 60%. Other findings of PAP include ptosis of the upper eyelid, decreased fullness of the inferior orbital fat pads, mild enophthalmos, orbital fat atrophy, flattening of the lower eyelid bags, and inferior scleral show [3,5]. The development of DUES and orbital fat atrophy can take from 1 month to 5 years [6]. Although PAP has been described in many cases, there is no consensus on its frequency and pathophysiology. Initially, the mechanism of PAP was associated with the function of Müller’s muscle, but a subsequent magnetic resonance imaging (MRI) study showed a decrease in orbital fat tissue after long-term use of PGAs [7]. Currently, most evidence suggests that PAP results from orbital fat atrophy due to adipogenesis inhibition and lipolysis induced by Prostaglandin F (FP) receptor stimulation [8].

Although there are objective evaluation methods for enophthalmos and ptosis, which are components of PAP, there is no examination method that encompasses all PAP changes as a whole. Therefore, subjective evaluation of the periocular region with or without photographic documentation is the primary clinical approach for diagnosis and follow-up [6]. Imaging is generally not used in PAP. However, due to unilateral findings in patients using PGAs in only one eye, computed tomography (CT) or MRI examinations may be utilized during the investigation of pseudoproptosis appearance that is usually present in the contralateral eye [7,9,10]. There are limited studies in the literature regarding the imaging findings of PAP patients. Although there are a few MRI studies [11,12], to our knowledge, there is no study conducted using CT. The ability of CT to image with sections thinner than 1 mm, its three-dimensional reconstruction capability, and post-scan multiaxial reformats suggest that CT could be supportive in imaging PAP findings. While it may not be used routinely in clinical practice to avoid radiation exposure, evaluating PAP findings in patients who undergo CT for other reasons could provide additional benefits.

In our study, we aim to retrospectively evaluate the thin-section CT scans of the orbital region taken for other reasons in patients using PGAs, investigate PAP-related findings, and compare them with the other eyes of the same patients not using PGAs.

## 2. Materials and Methods

Ethics committee approval was obtained for this study (EBYU KAEK 2024-05/03). The principles of the Helsinki Declaration were adhered to during this study. Due to the retrospective design of this study, additional informed consent was not required from patients beyond the general information and consent form routinely obtained prior to imaging procedures for the scientific use of the images.

### 2.1. Patient Selection

This study includes patients with open-angle glaucoma who use PGA in one eye and have available orbital region CT images. Inclusion criteria were defined as unilateral use of PGA and availability of orbital CT images obtained at least 1 month after the initiation of PGA treatment. To exclude conditions that could cause enophthalmos or exophthalmos, exclusion criteria included the following: imaging due to trauma, presence of masses in the orbital region, history of orbital inflammatory disease or thyroid orbitopathy, strabismus surgery, scleral buckling, oculoplastic or orbital wall surgery. Additionally, >2 diopters of refractive error and artifacts in CT images that prevent evaluation were also exclusion criteria.

### 2.2. Ophthalmology and Radiology Evaluation

Data on patients’ age, sex, ophthalmological examination characteristics, refraction measurements, type of glaucoma, type of PGA used, duration of PGA use until the CT scan, total number of medications, and information about antiglaucomatous medications other than PGA were retrospectively collected from patient records. Orbital CT images were reviewed retrospectively from the hospital information management system. The contralateral eyes of the same patients not using PGAs served as the control group.

**Figure 1:**
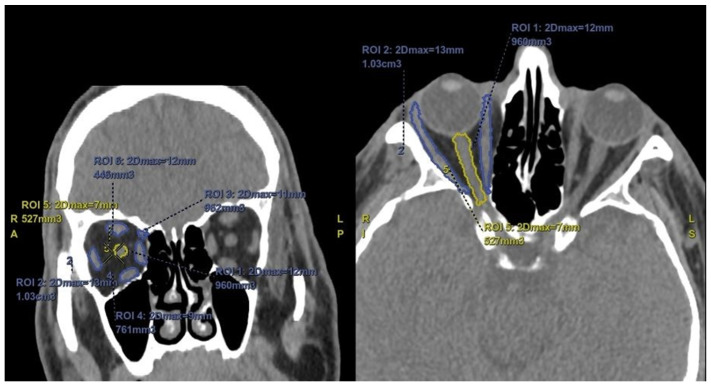
Coronal and axial sections with manual region of interest (ROI) for muscle and optic nerve volume measurements.

Two radiologists with 10 years and 11 years of experience performed the radiological evaluations of the patients, blinded to their clinical characteristics. The measurements of the first radiologist were accepted as the study data. To test the repeatability of the measurements, a second radiologist with 11 years of experience repeated the same measurements. Enophthalmos measurements were performed on axial section images of the CT. For this evaluation, an interzygomatic horizontal line was drawn on the axial section, and the distances of the corneal edge to this horizontal line were measured anteriorly. Additionally, volume measurements of the orbital fat, periorbital muscles, and optic nerve were performed. Using the software on the workstation (AW Server 4.7 by GE Healthcare, Milwaukee, WI, USA), the radiologists manually marked the free regions of interest (ROI) for orbital fat tissue, periorbital muscles, and optic nerve separately in both eyes on sagittal, coronal, and axial sections, and three-dimensional volume measurements were performed (Figure 1).

### 2.3. Statistical Analysis

A sample size calculation was performed. Using the G*Power 3.1.9.7 software, a power analysis was conducted with an α error probability of 0.05 and a power of 0.80, indicating that a minimum of 51 patients per group would be required to achieve sufficient statistical power. However, due to the stringent exclusion criteria, we fell slightly short of the 50 patients we aimed for to achieve 80% power. Nevertheless, we conducted a post hoc analysis with the current number of patients. A post hoc power analysis was conducted using G*Power software, which yielded a power of 0.716. While this value is slightly below the commonly accepted threshold of 0.80, it still indicates a moderately strong ability to detect significant effects in the sample.

The patients’ data were analyzed using the Statistical Package for Social Sciences (SPSS) for Windows 20 software (IBM SPSS Inc., Chicago, IL, USA). Descriptive statistics such as mean, standard deviation (SD), and ratio values were used to describe the data. The normality of the data distribution was assessed using the Shapiro–Wilk test. For the analysis of numerical dependent variables that did not show a normal distribution, the Wilcoxon test was used. The Kruskal–Wallis test was applied to compare CT measurements among the three PGA types. Spearman correlation analyses were applied to assess correlations between variables. Intraclass correlation coefficients (ICCs) were used to evaluate the reliability of the measurements between the two evaluators. An ICC between 0.75 and 1.0 was considered excellent, while an ICC between 0.40 and 0.75 was considered fairly good [13]. With the Bland–Altman analysis, the agreement of the differences between the measurements was evaluated and Bland–Altman plots were generated to visualize the differences between the two measurements. A value of *p* < 0.05 was considered statistically significant.

## 3. Results

Forty patients with a history of using PGA in one eye for at least 1 month prior to the CT scan were included in the study. Of these patients, 19 (47.5%) were female and 21 (52.5%) were male. The average age was 68.48 ± 11.69 years. Seventeen patients (42.5%) had no systemic disease, while 23 patients (57.5%) had one or more systemic diseases (diabetes mellitus, hypertension, coronary artery disease). All patients had open-angle glaucoma; 30 patients (75%) had primary open-angle glaucoma (POAG) and 10 patients (25%) had pseudoexfoliation glaucoma. Twenty-nine patients (72.5%) were using latanoprost, 9 patients (22.5%) were using bimatoprost, and 2 patients (5%) were using travoprost (Table 1). The average duration of PGA use before the CT scan was 35.88 ± 55.04 months (range: 2–272 months). Fourteen patients (35%) were using PGA monotherapy, while 26 patients (65%) were using one or more additional antiglaucomatous treatments. The total number of medications and treatment details for the patients can be seen in Table 1. Additionally, out of the 26 patients using combination therapy, 5 (19.2%) used the fixed combination of PGA and beta-blocker, while 21 (80.8%) used PGA and beta-blockers separately.

**Figure 2:**
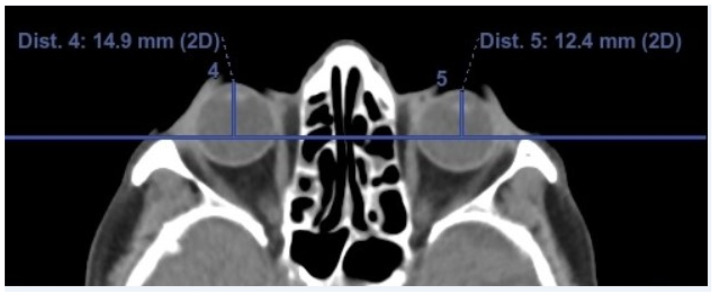
Enophthalmos measurements of a patient using a latanoprost–timolol fixed combination in the left eye for 24 months show a difference of more than 2 mm between the two eyes.

The average enophthalmos measurements and tissue volumes obtained from the CT images are shown in Table 2. Accordingly, the mean enophthalmos values on the side treated with PGA were found to be slightly lower compared to the untreated side; however, this difference did not reach statistical significance (15.5 ± 2.0 mm vs. 16.1 ± 1.4 mm, respectively) (*p* = 0.07). In 29 patients (72.5%), the enophthalmos measurements on the treated side were smaller compared to the untreated side, with a difference of 2 mm or more in 7 patients (17.5%) (Figure 2). 

**Figure 3:**
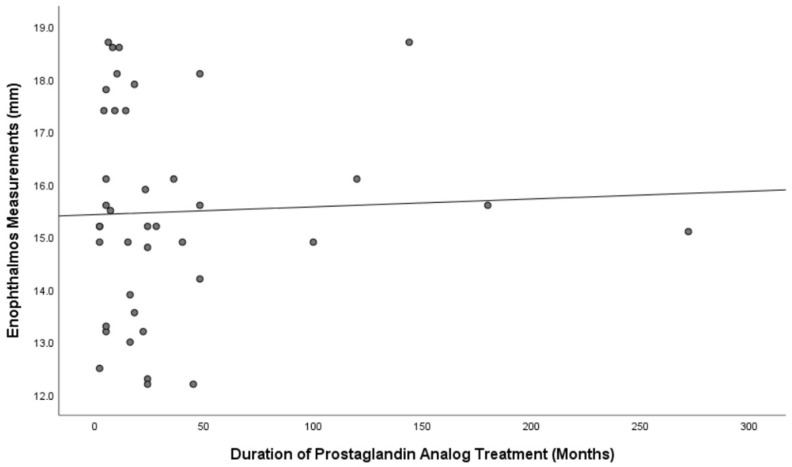
Correlation graph between the duration of prostaglandin analog treatment and enophthalmos. There was no significant correlation between the duration of prostaglandin analog treatment and enophthalmos (*p* = 0.768, r = −0.048).

There was no significant correlation between the duration of PGA use and the enophthalmos measurements (*p* = 0.768 r = −0.048) (Figure 3). In the correlation analysis conducted to determine whether changes in the results were related to age, no significant correlation was found between enophthalmos measurements and age (*p* = 0.118, r = −0.251). In the subgroup analysis for latanoprost and bimatoprost, no correlation was found between medication use duration and enophthalmos measurements in either group (*p* > 0.05), excluding the travoprost group due to a small sample size. Among the seven patients with a posterior displacement of 2 mm or more between the eyes, one was using bimatoprost (duration: 5 months), one was using travoprost (duration: 11 months), and five were using latanoprost. The mean duration of latanoprost use among these patients was 24.6 ± 11.9 months (range: 16–45 months). No significant differences were found between the groups in terms of orbital fat volume, total extraocular muscle volume, and optic nerve volume (*p* > 0.05) (Table 2). In the comparison of the three subtypes of PGAs included in this study, no significant differences were found within the treated eyes or within the control eyes regarding enophthalmos measurement, orbital fat volume, total extraocular muscle volume, and optic nerve volume (all *p* > 0.05).

**Figure 4:**
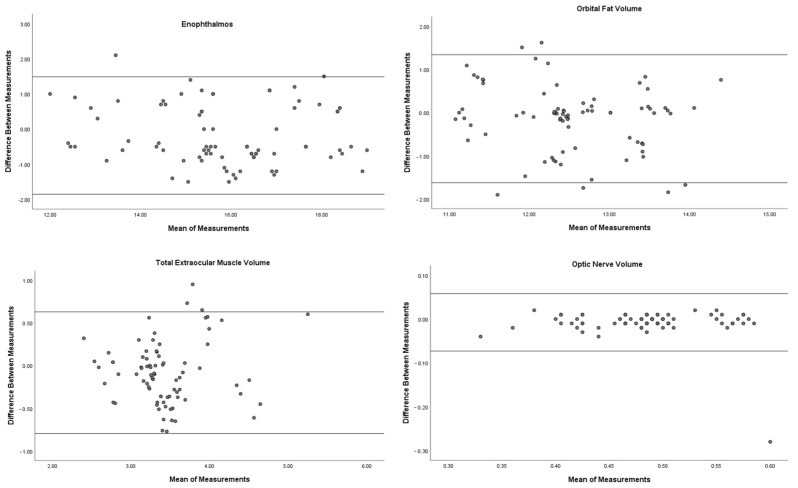
The Bland–Altman plots demonstrate the measurements of enophthalmos, orbital fat volume, total extraocular muscle volume, and optic nerve volume. In the graphs, the upper line represents the “Upper Limit of Agreement”, and the lower line represents the “Lower Limit of Agreement”.

Although relatively lower ICC values were obtained in orbital fat and total extraocular muscles measurements, excellent reliability was observed in all measurements with ICC values exceeding 0.75 (Table 3). To assess the validity and statistical significance of the model on all measurements separately, we used “ANOVA with Tukey’s Test for Nonadditivity” and the “Hotelling’s T-Squared Test”, which yielded a *p*-value > 0.05, indicating that these results were not statistically significant. However, the “F Test with True Value 0” showed a *p*-value of <0.001, confirming the accuracy of the agreement between the measurements. Additionally, the Bland–Altman plots created to visualize the differences between the two measurements are shown in Figure 4.

## 4. Discussion

The primary diagnosis of PAP is based on history and clinical examination, and generally does not require laboratory tests or imaging unless ruling out other pathologies. However, PAP findings can lead to changes detectable via imaging methods [7,10]. Although CT and MRI have limited capacity to detect small changes in the anterior segment structures, observing decreased orbital and periorbital fat volume without inflammatory changes on orbital, maxillofacial, and cranial CT or MRI is important, especially in unilateral cases, to rule out conditions that can result in enophthalmos, such as silent sinus syndrome, or to exclude presumed orbital pathologies in the contralateral eye.

In our study, no significant difference was found between the average enophthalmos values of the treated and untreated sides in patients using PGA. However, in 72.5% of the patients, the enophthalmos measurements were lower on the treated side compared to the untreated side, and in 17.5% of the patients, the posterior displacement was 2 mm or more. Although a few millimeters of enophthalmos may not always be clinically noticeable, it can cause difficulties in IOP measurement, challenges during anterior segment surgery, and cosmetic concerns [10,14]. Although no study on CT findings in patients using PGA was found in the literature, Filippopoulos et al. presented orbital CT images of a patient who used bimatoprost in the right eye for 4 years and presented with fullness in the left upper eyelid to rule out exophthalmos in the left eye. The CT images showed a normal orbit on the left and a more posteriorly positioned globe on the right [10]. In another study, enophthalmos was detected in 80% of patients using bimatoprost, 35% of patients using travoprost, and 7.1% of patients using latanoprost, as measured using a Hertel exophthalmometer [6]. In a study assessing glaucoma patients treated with bimatoprost using Hertel exophthalmometry, the mean Hertel value was found to be 11.9 mm, and it was highlighted that this value was suspicious for enophthalmos [15]. However, cases with symmetrical globes despite long-term PGA use have also been reported in the literature [10]. Additionally, the limitations of Hertel exophthalmometry include its dependency on the evaluator and orbital anatomy. 

Imaging methods can yield more objective results for the assessment of enophthalmos. In the literature, a case report exists where a patient with a history of bimatoprost use in the left eye had 2 mm of relative enophthalmos detected with a Hertel exophthalmometer, and enophthalmos and kinking of the left optic nerve were seen on MRI [10]. In a study using MRI to measure enophthalmos in nine glaucoma patients using bimatoprost, Higashiyama et al. measured enophthalmos using an axial section baseline drawn from the zygomatic bone’s frontal process, similar to our study, and found a significant difference between the treated and contralateral sides (14.7 ± 2.5 vs. 16.0 ± 2.3 mm, respectively) [11]. The difference between our study and the MRI study may be due to the different types of PGAs used by the patients in the two studies.

The type of PGA used is a factor affecting the incidence of PAP. The first report related to DUES was associated with bimatoprost use, followed by additional reports related to travoprost [4,16]. Different studies in the literature report that PAP development is most common in patients using bimatoprost (60%), followed by travoprost (53%) and latanoprost (approximately 6%) [17,18,19]. In their study examining the factors related to PAP development, Patradul et al. found that advanced age, bimatoprost and travoprost use, and PGA-timolol fixed combination use were risk factors for PAP development [20]. Additionally, it has been reported that 60% of patients who switched from latanoprost to bimatoprost developed DUES three months after the change in treatment [17]. In our study, 72.5% of the patients were using latanoprost, the most commonly used PGA, and 19.2% of patients using multiple antiglaucomatous medications were taking a fixed combination of PGA and timolol. Although the risk of PAP development with latanoprost use is lower compared to bimatoprost and travoprost, long-term unilateral latanoprost use has been reported to cause PAP [21,22,23]. In our study among patients with a difference of more than 2 mm in enophthalmos measurements between the two eyes, those using latanoprost had a mean duration of 24 months of use.

In our study, no significant difference was found in orbital fat volume between the treated and contralateral sides. However, Jayaprakasam et al. reported that MRI of a patient who used bimatoprost in the right eye for 2 years and presented with progressive fullness in the left eyelid revealed no cause for proptosis in the left eye but showed less periorbital fat tissue in the right eye, with the fat loss being more pronounced in the medial orbit [7]. In an MRI study comparing 23 POAG patients treated with PGA and 21 healthy controls, Chen et al. found a decrease in orbital fat volume from the orbital apex to the trochlea in the glaucoma group but reported that this difference was due to posterior orbital fat tissue (between the orbital apex and the globe-optic nerve junction). They found similar fat volumes in the anterior region (between the trochlea and the globe–optic nerve junction) and suggested that their findings were unlikely due to PGA use because of the topical application method [12]. Another MRI study on patients using bimatoprost in one eye reported significantly lower mean orbital fat volume on the treated side compared to the untreated side [11]. PGAs have previously been reported to inhibit adipogenesis via FP receptor stimulation [8]. In line with this, an in vitro study demonstrated that PGAs inhibit preadipocyte differentiation and intracellular lipid accumulation [24]. Additionally, Park et al. compared average adipocyte density with preaponeurotic orbital fat biopsy in patients using PGA and found no difference in the latanoprost group, while the bimatoprost and travoprost groups had higher adipocyte densities compared with the control groups. They also noted pathological findings suggestive of fat atrophy, such as clustering of adipocyte nuclei, in eyes treated with PGAs. The authors proposed that this fat atrophy could be a mechanism for DUES development in patients using topical PGAs [25]. The difference between our study and the existing studies may be related to the fact that the majority of our patient population used latanoprost.

Another factor discussed regarding the development of PAP is the duration of PGA use. There are studies in the literature that did not find a significant relationship between the duration of PGA use and the development of PAP [20,23]. However, many prospective studies investigating the risk of PAP development have found that the incidence of PAP increases with longer PGA use (at 1, 3, and 6 months) [17,18]. In their study, Küçükevcilioğlu et al. reported the earliest onset of PAP as 4 months for latanoprost, 2 months for bimatoprost, and 1 month for travoprost [6]. Due to the retrospective cross-sectional design of our study, it was not possible to prospectively evaluate the effect of PGA duration on enophthalmos and orbital fat volume. However, we did not find a significant correlation between the duration of PGA use before CT and the enophthalmos measurements.

Other factors discussed regarding the development of PAP are age and gender. While some studies did not find a relationship between demographic factors and the development of PAP [23,26], others identified age as a risk factor [17]. Maruyama et al. reported that the incidence of DUES was significantly higher in older patients, but it was not associated with gender, refraction, or IOP levels [18]. Patradul et al. identified being over 60 years old as a risk factor for PAP development [20]. The fact that almost all of our patients were elderly prevented us from performing a subgroup analysis for age in our measurements. Using the contralateral eyes of the patients not using PGAs as the control group eliminated the difficulty of distinguishing changes due to aging from those due to PGA use.

Since the reliability of any measurement or evaluation tool must be investigated before it is used in research or clinical practice [27], we analyzed inter-rater correlation coefficients for all measurements performed in our study and found excellent reliability across all measurements. In a previous study evaluating patients who used PGA in one eye with MRI, volume measurements of the orbital tissues were conducted with only a single measurement, and ICC was not calculated [11].

Our study has some limitations. Firstly, PAP is a clinical term with many components, and although we evaluated the orbital fat volume and the axial position of the globe with CT, clinical evaluations such as DUES, dermatochalasis, and scleral show, which are part of PAP, were not included in the study. Another limitation is the relatively small sample size of the study. Additionally, the retrospective design did not allow us to obtain CT images from the period before PGA use to measure orbital fat tissue and enophthalmos. Many patients in our study using multiple glaucoma treatments was another limitation. The concurrent use of multiple medications alongside PGA may potentially confound the development of PAP. Some studies in the literature suggest associations between the use of PGA in combination with beta-blockers or carbonic anhydrase inhibitors and the development of PAP [20,28]; however, research in this area is limited.

## 5. Conclusions

In conclusion, we did not find significant differences in enophthalmos measurements, orbital fat volume, total muscle volume, and optic nerve volume between the eyes treated with PGA and the untreated eyes. However, the changes observed in some of the patients suggest the need for longitudinal prospective studies with larger patient populations and an evaluation of different PGA subgroups.

## Figures and Tables

**Table 1:** Demographic and ophthalmological characteristics of patients.

Demographic and Ophthalmological Characteristics	Patients Receiving PGA Treatment (n = 40)
Age (Years), Mean ± SD	68.4 ± 11.6
Sex, n (%) Female Male	21 (52.5%) 19 (47.5%)
Type of Glaucoma, n (%) POAG PXF Glaucoma	30 (75.0%) 10 (25.0%)
Duration of PGA treatment * (Months), Mean ± SD	35.8 ± 55.0
Number of Antiglaucomatous Medications, Mean ± SD	3.0 ± 1.3
Type of Antiglaucomatous Treatments, n (%) PGA Monotherapy Combined PGA Treatment • PGA + BBs + CAIs + α-agonists • PGA + BBs + CAIs • PGA + BBs + α-agonists	14 (35.0%) 26 (65.0%) 19 (47.5%) 4 (10.0%) 3 (7.5%)
Type of PGA Treatment, n (%) Latanoprost Bimatoprost Travoprost	29 (72.5%) 9 (22.5%) 2 (5.0%)

PGA: prostaglandin analogs, POAG: primary open-angle glaucoma, PXF: Pseudoexfoliation, BBs: beta-blockers, CAIs: carbonic anhydrase inhibitors. * prior to CT imaging.

**Table 2:** Computed tomography (CT) measurement results according to prostaglandin analogs (PGA) treatment.

CT Measurements	PGA Treated Side (n = 40)	PGA Untreated Side (n = 40)	*p* Value
Enophthalmos (mm)	15.5 ± 2.0	16.1 ± 1.4	0.070
Orbital Fat (cm^3^)	12.3 ± 0.8	12.5 ± 0.7	0.180
Total Extraocular Muscles (cm^3^)	3.3 ± 0.4	3.4 ± 0.6	0.814
Optic Nerve (cm^3^)	0.4 ± 0.1	0.4 ± 0.1	0.135

CT: Computed tomography, PGA: prostaglandin analogs.

**Table 3:** Intraclass correlation coefficients (ICC) for computed tomography measurements.

CT Measurements	Cronbach’s α Coefficient	Intraclass Correlation Coefficient	95% Confidence Interval	
			Lower Bound	Upper Bound
Enophthalmos	0.938	0.882	0.822	0.923
Orbital Fat	0.777	0.772	0.646	0.854
Total Extraocular Muscles	0.860	0.755	0.642	0.836
Optic Nerve	0.904	0.825	0.740	0.884

CT: Computed tomography.

## Data Availability

The data presented in this study are available on request from the corresponding author. The data are not publicly available due to ethical restrictions.
